# Updated modelling of the prevalence of immunodeficiency-associated long-term vaccine-derived poliovirus (iVDPV) excreters

**DOI:** 10.1017/S095026881900181X

**Published:** 2019-10-24

**Authors:** D. A. Kalkowska, M. A. Pallansch, K. M. Thompson

**Affiliations:** 1 Kid Risk, Inc., Orlando, USA; 2 Division of Viral Diseases, National Center for Immunization and Respiratory Diseases, Centers for Disease Control and Prevention, Atlanta, GA, USA

**Keywords:** Polio, polio vaccine virus, virology (human) and epidemiology

## Abstract

Conditions and evidence continue to evolve related to the prediction of the prevalence of immunodeficiency-associated long-term vaccine-derived poliovirus (iVDPV) excreters, which affect assumptions related to forecasting risks and evaluating potential risk management options. Multiple recent reviews provided information about individual iVDPV excreters, but inconsistencies among the reviews raise some challenges. This analysis revisits the available evidence related to iVDPV excreters and provides updated model estimates that can support future risk management decisions. The results suggest that the prevalence of iVDPV excreters remains highly uncertain and variable, but generally confirms the importance of managing the risks associated with iVDPV excreters throughout the polio endgame in the context of successful cessation of all oral poliovirus vaccine use.

## Introduction

As part of its effort to eliminate the risks of poliomyelitis disease, the Global Polio Eradication Initiative (GPEI) began a phased process of cessation of oral poliovirus vaccine (OPV) use, starting with the removal of serotype 2 OPV in April–May 2016 [1]. The GPEI Strategic Plan 2013–2018 [2], which the GPEI extended to 2019 [3], calls for coordinated cessation of all OPV serotypes following the global certification of elimination of the remaining two wild poliovirus (WPV) serotypes (i.e. 1 and 3). By reducing exposure to OPV, the risk of vaccine-associated paralytic poliomyelitis (VAPP) decreases, which occurs in a small fraction of OPV recipients and their close contacts [4]. Successfully ending OPV use also limits the possibility of creating future circulating vaccine-derived polioviruses (cVDPVs) that can behave like WPVs in settings with low-population immunity to transmission [4–6]. Finally, stopping OPV prevents new live poliovirus infections in the small number of individuals with some B-cell-related primary immunodeficiency diseases (PIDs) who do not clear poliovirus infections or take significantly longer to clear poliovirus infections compared to individuals with competent immune systems, which we refer to as immunodeficiency-associated long-term vaccine-derived poliovirus (iVDPV) excreters. [7]. While immunocompetent individuals infected with poliovirus excrete on average for around 30 days but no longer than 3 months [5, 8–10], iVDPV excreters can shed virus for variable and substantially longer periods of time [11–15]. Consequently, individual iVDPV excreters may increase the risks of re-introducing and re-starting live poliovirus transmission in populations, which becomes more recognisable after other transmission of live polioviruses stop [4, 7, 16, 17].

While OPV cessation of any serotype leads to the direct and immediate result of discontinuation of the incidence of recipient VAPP cases of that serotype in immunocompetent individuals, the evolution of OPV-related strains circulating in low-immunity populations can take months until they become apparent as cVDPVs [6]. In addition, individuals in a population can become exposed to OPV excreted by vaccine recipients, and in rare instances develop contact VAPP if not already protected by prior exposure or vaccination [4]. Individuals infected with polio can present clinically with VAPP at any time during their infections. Thus, during the time that they remain infected, iVDPV excreters can develop VAPP (iVAPP).

Several recent reviews provided information about individual iVDPV excreters [18–20]. These reviews provide details about the individual cases that they included, however, some inconsistencies exist between the reviews (e.g. inclusion or exclusion of some individuals in some reviews but not others), and in many instances key data gaps remain (e.g. missing information about the nature of the immunodeficiency, which affects all reviews). With the increased use of poliovirus environmental surveillance, the detection of virus likely excreted by iVDPV excreters into catchment areas covered by environmental surveillance also creates a category of potential non-identified iVDPV excreters [4, 7, 21–27].

A prior stochastic, discrete-event simulation (DES) model provided estimates of the global prevalence of long-term iVDPV excreters based on the best available evidence as of 2014 [28]. This model supported a global integrated analysis that suggested iVDPV excreters may present a significant risk in the polio endgame, along with any OPV (un)intentionally remaining in the field or inadequately contained in laboratories and vaccine production facilities [29]. In the post-OPV cessation era, inactivated poliovirus vaccine (IPV) remains the only option for immunisation against polio [30]. However, while vaccination with IPV protects vaccine recipients from paralysis if they later become infected by a live poliovirus, IPV does not prevent or stop transmission in most populations (i.e. those characterised by predominantly faecal–oral transmission) [8, 9]. For example, serotype 1 WPV (WPV1) circulated successfully in Israel for a year despite its very high coverage with IPV in routine immunisation [31, 32]. With the cessation of OPV use, population immunity to poliovirus transmission continues to decline as more time passes since OPV cessation, which further exacerbates the inability of IPV to provide sufficient population immunity to prevent or stop potential transmission [7, 28, 33, 34].

The GPEI partners recognised the risk posed by long-term iVDPV excreters and the limited tools available to prevent or to stop future iVDPV-associated outbreaks, which motivated research and development of polio antiviral drugs (PAVDs) [35]. Application of the prior DES model considering different assumptions about patient screening and potential PAVD availability and effectiveness helped to support the economic case for the development of PAVDs and PID screening [7, 16, 17]. To date, the first drug (pocapavir) performed well in clinical studies (phases 1 and 2) with respect to safety and reduction of viral excretion [36, 37]. However, the use of pocapavir also led to the emergence of drug-resistant strains in the study site setting, and a second PAVD that works by a different mechanism is being developed for use in combination with pocapavir. This updated assessment of future iVDPV risks considers the accumulation of new evidence about the prevalence of individuals with specific B-cell related PIDs relevant to prolonged and chronic poliovirus infections, survival rates, and treatment with intravenous immunoglobulin (IVIG) therapy, as well as increased screening of PID patients for long-term iVDPV excretion and delays in the expected availability of PAVDs.

## Methods

### Synthesis of evidence related to PID patients and iVDPV excretion

The global PID prevalence remains highly uncertain and dynamic due to the large number of PID conditions, differences in case definitions, increased detection and variability between countries in genetic profiles and survival rates of PID patients [38–40]. Some information on PID occurrence comes from surveys among physicians who participate in the Jeffrey Modell Foundation Network (JMFN), a network created and maintained by a private foundation that focuses on identifying and getting treatment for individuals with PIDs (see Table 1) [41–44]. Based on the surveys performed this decade, the estimated number of PID patients increased from ~60 000 PID patients in 2011 to ~94 000 PID patients in 2018 [41–44]. However, the inconsistent and incomplete nature of the data collected from these surveys makes it difficult to estimate the actual rate of increase in the number of PID patients (e.g. the number of countries covered increased, and the numbers of physicians and/or centres in the network and the size of survey response changed over time (see Table 1) [41–44]). Nonetheless, these data provide the only, albeit limited, evidence relevant to the estimation of the number of PIDs globally. Although the JMFN surveys report PID prevalence stratified by different criteria and type of PID, we focus on the subset of PID defects for which known long-term poliovirus excreters exist(ed) (i.e. common variable immunodeficiency disease (CVID) and other PIDs with B-cell involvement relevant to long-term poliovirus excretion (oPIDs), including but not limited to severe combined immunodeficiency disease (SCID), hypogammaglobulinemia (HGG), major histocompatibility complex (MHC) Class II and X-linked agammaglobulinemia (XLA)). Using this subset, we fit the model to produce global CVID and oPID prevalence estimates consistent with the known CVID and oPID prevalence, which we assume provides a reasonable basis for further estimating the prevalence of long-term iVDPV excreters.

**Table 1.** JMFN surveys and data on patients with PIDs over time [41–44]

Number of	2011	2013	2015	2018
Countries covered	64	78	84	86
Physicians in JMFN	490	556	602	792
Centres in JMFN	254	234	253	358
Surveys returned	192	225	225^a^	252^a^
Followed patients	79 764	138 847	157 454	187 988
Diagnosed patients	60 364	77 193	83 743	94 024
Treated patients (IVIG)^b^	14 140	17 225	20 427	23 967
CVID	7613	8582	10 545	11 996
oPIDs^c^	12 073	15 439	16 749	18 805

CVID, common variable immune deficiency; JMFN, Jeffrey Modell Foundation Network; IVIG, intravenous immunoglobulin; oPID, other PIDs; PID, primary immune deficiency.

a Number of returned surveys in 2015 and 2018 estimated based on description.

b Treatment includes delivery of IVIG therapy and any other appropriate clinical services, note that treatment does not effectively stop existing excretion.

c oPID defects for which known long-term poliovirus excreters exist(ed), estimated as 20% of all diagnosed patients.

We maintain a database of iVDPV excreters (first discussed in a 2006 paper [4] and later updated in 2015 [7], but not published in 2015 as an individual line listing). With respect to known individual iVDPV excreters, we updated our database using information obtained from the published literature and personal communications. We added fields to our database to support a cross comparison of the evidence available from different sources. We use prior assessments of iVDPV risks that characterise excreters as chronic if their excretion exceeded 5 years, and prolonged for those excreting at least 6 months but less than 5 years [4]. The most recent published registry of long-term excreters known to the World Health Organisation (WHO) consists of 101 individuals (94 prolonged and 7 chronic) identified between 1962 and 2016, 21 (21%) diagnosed with CVID and the remainder diagnosed with oPIDs [19]. In that review, 7 of the 21 (33%) long-term excreters with CVID and none of the long-term excreters with oPIDs met the criterion as chronic excreters [19]. Since the time of publication of that review [19], multiple new long-term excreters have been described [45, 46]. A 2018 systematic review [20] reported 107 individual iVDPV excreters, 93 of which we matched to entries in the WHO review [19]. We also found eight iVDPV excreters in the WHO review [19] missing from the 2018 systematic review [20], and 14 new iVDPV excreters identified by the 2018 systematic review [20] not in the WHO review (some that occurred after its publication) [19]. We included information provided through personal communications with the WHO, which continues to update its registry, and with individuals who reported on specific cases in the literature. We also verified that all of the information that we reported for USA cases in our database contained correct information according to Centers for Disease Control and Prevention (CDC) archives. Our database also includes a list of environmental isolations of ambiguous VDPVs (aVDPVs) that suggest the presence of prolonged and chronic excretion by unidentified iVDPV excreters. The data we present here reflect the information in our database as of 1 May 2019.

### Modelling

**Fig. 1.**
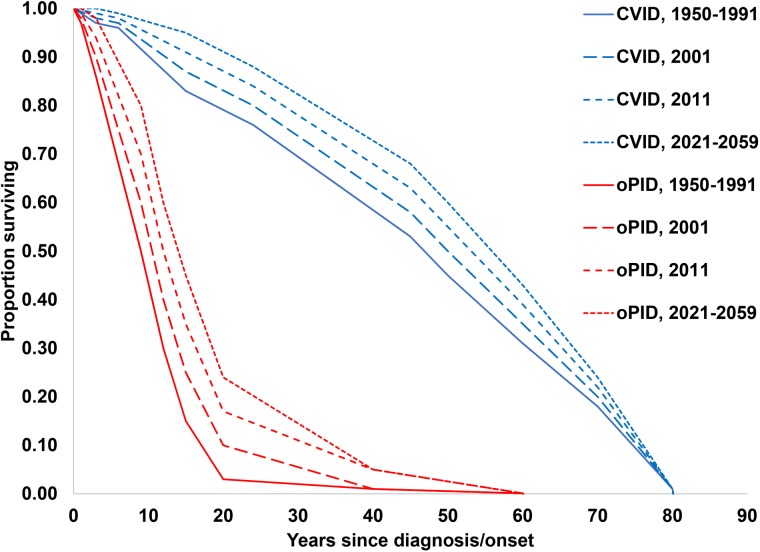
Assumed baseline survival curves for CVID and oPID patients effectively-treated (with IVIG) in a population with *R*_0_ values for WPV1 of 4 or 5.

**Fig. 2.**
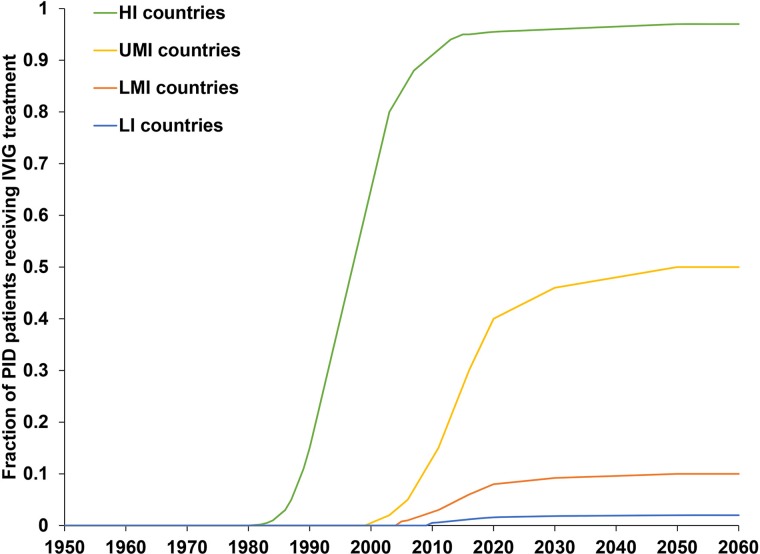
Assumed fractions of CVID and oPID patients treated with IVIG as a function of time, by income level.

The previously-developed DES model tracks how CVID and oPID patients move through various clinical and OPV infection stages using a discrete time step of 1 month to estimate long-term poliovirus excreter prevalence over time following each modelled individual for life [7]. The model accounts for different characteristics of transmission and vaccine schedules by stratifying the global population into blocks. We updated the approach that we use [16] to stratify the world into 72 epidemiological blocks of 107 million people each (corresponding to the global population of 7.2 billion as of 2018). We updated the characterisation of the blocks to use the 2018 World Bank income level (i.e. low-income (LI), lower middle-income (LMI), upper middle-income (UMI), high-income (HI)) [47] and polio vaccine use (i.e. use of OPV, role of IPV). For each block, based on prior modelling experience we assumed a basic reproduction number (*R*_0_) to account for many factors that affect poliovirus transmission and the health system quality [7]. We report the *R*_0_ for WPV1 and then the model applies fixed relative ratios to compute the *R*_0_ for serotype 2 WPV (WPV2) and serotype 3 WPV (WPV3) relative to the *R*_0_ for WPV1 [7]. Table 2 presents the updated inputs for the DES model describing births and attributes assigned at birth, deaths and monthly event probabilities and related relative probabilities (i.e. CVID or oPID onset, diagnosis, OPV infections and iVAPP). With increasing adoption of medical technology globally [48], we updated our assumptions about the delay in PID diagnosis probabilities by the income level for both CVIDs and oPIDs. Similarly, in view of improvements in medicine and changing standards of care over time, we updated our assumptions about patient survival and access to treatment. Specifically, considering a new study that suggested a higher rate of CVID patient survival [49] compared to the earlier estimates [50], we assumed a higher proportion of surviving CVID and oPID patients relative to their year of birth (i.e. improvement over time). Figure 1 shows the updated assumed baseline survival curves for effectively-treated (with IVIG) CVID and oPID patients over time. As in the prior model [7], we assume lower treatment fractions in lower income levels, but we assume an increase in treatment fractions over time, with higher projected values compared to our previous estimates. Figure 2 shows the updated assumed treatment fraction as a function of time for each income level.

**Table 2.** Inputs for the updated DES model of long-term poliovirus excreter prevalence [7]

Model input	Value
*Births and probabilities of attributes determined at birth*	
Births, by income level and polio vaccine use as of 2018 [1/month]	Varies with time
PID pre-disposition	
CVID	1/32 000
oPIDs	1/8500
Potential long-term excretion (if OPV-infected and surviving)	
Prolonged, CVID or oPIDs	0.01
Chronic, CVID	0.005
Chronic, oPID	0
*Deaths*	
Death, general population, by income level	Varies with age and country
Death, CVID or oPID patients	Varies with time since onset/treatment
Relative monthly death rate *vs.* baseline, by treatment status	
Treated	1
Untreated	5
Relative monthly death rate *vs.* baseline, by *R*_0_ for WPV1	
4 or 5	1
6	5
7	10
8	20
9	25
10	35
11	40
12	45
13	50
*Monthly event probabilities and related relative probabilities*	
PID onset	
CVID	1/300
oPIDs	1/24
CVID diagnosis	
LI	1/240
LMI	1/120
UMI	1/60
HI	1/36
oPID diagnosis	
LI	1/60
LMI	1/12
UMI	1/5
HI	1/6
Treatment lapse	
LI	0.8
LMI	0.75
UMI	0.1
HI	0.001
Primary OPV infection, if OPV-only RI and undiagnosed CVID or oPID	
Any income level, age 0	1/4
Not high-income level, age 1–4	1/12
High-income level, age 1–4	1/48
Any income, age >4	0
Primary OPV infection, if IPV/OPV RI and undiagnosed CVID or oPID	
Any income level, age 0	1/6
Any income level, age 1–4	1/48
Any income, age >4	0
Relative probability of primary OPV infection, diagnosed *vs.* not diagnosed	0.1
Relative probability of secondary OPV infection, diagnosed *vs.* not diagnosed	0.5
Secondary OPV infection, if OPV-only RI	
Not high-income, age 0–4	1/24
High-income, age 0–4	0.029
Not high-income, age 5–14	0.5 × 1/24
High-income, age 5–14	0.5 × 0.029
Not high-income, age >14	0.25 × 1/24
High-income, age >14	0.25 × 0.029
Relative probability of secondary OPV infection in any income level if IPV/OPV RI *vs.* high-income country with OPV-only RI	0.5
OPV cessation	
Serotype 1 (assumed)	1 January 2025
Serotype 2	1 May 2016
Serotype 3 (assumed)	1 January 2025
Serotype-specific OPV infection given any OPV infection, before OPV2 cessation	
Serotype 1	0.135
Serotype 2	0.658
Serotype 3	0.144
Serotype 1 and 2	0.027
Serotype 1 and 3	0
Serotype 2 and 3	0.036
Serotype 1 and 2 and 3	0
Serotype-specific OPV infection given any OPV infection, after OPV2 cessation	
Serotype 1	0.50
Serotype 2	0
Serotype 3	0.50
Serotype 1 and 2	0
Serotype 1 and 3	0
Serotype 2 and 3	0
Serotype 1 and 2 and 3	0
Relative probability of long-term OPV infection if treated with IVIG *vs.* not treated	0.5
Recovery from OPV infection, by time since onset of infection	
Typical, months 0–4	1/3
Typical, month 5	1
Prolonged, month 0–5	0
Prolonged, months 6–58	1/24
Prolonged, month 59	1
Chronic, month 0–59	0
Chronic, from month 60	1/180
iVAPP	
CVID, untreated	0.004
oPID, untreated	0.008
Any CVID or oPID, treated with IVIG	0
Fatal iVAPP	
LOW	0.5
LMI	0.4
UMI	0.3
HIGH	0.14

CVID, common variable immune deficiency; HI, high-income; IPV, inactivated poliovirus vaccine; iVDPV, immunodeficiency-related vaccine-derived poliovirus; LI, low-income countries; LMI, lower middle-income; OPV, oral poliovirus vaccine; OPV2, serotype-2-containing OPV; oPID, other PID with B-cell involvement relevant to long-term poliovirus excretion; PID, primary immune deficiency; *R*_0_, average annual basic reproduction number; RI, routine immunisation; UMI, upper middle-income; iVAPP, vaccine-associated paralytic polio in immunodeficient individuals.

To run the model, we generate the number of births over time on a monthly basis for each block, based on demographic data [7]. We generate the expected number of newborns with a genetic PID predisposition relevant to polio long-term excretion (i.e. a future CVID or oPID) in each month using a random draw from a Poisson distribution with a rate equal to the number of birth times the fraction of births with PIDs. We implicitly assume that the inputs for pre-disposition for CVID and oPID average over any variability that exists in the rate of B-cell immunodeficiencies that result from consanguineous marriage and other risk factors and that these risk factors do not change with time. For each generated CVID or oPID pre-disposed individual at birth, we randomly determine whether he or she will become a long-term excreter after the onset of clinical symptoms if infected with a live poliovirus. On a monthly basis, for each such individual we check whether death occurs prior to clinical PID onset according to age-specific general population death rates for each income level [7]. Once clinical CVID or oPID onset occurs, we assume different monthly probabilities of death according to the CVID or oPID survival curves shown in Figure 1, IVIG treatment status (with the possibility of treatment lapse) and block-specific *R*_0_ values for WPV1. For all surviving CVID or oPID patients, we perform a monthly check to see whether clinical onset of the PID occurs in the model, and if it occurs, we introduce treatment (according to the probabilities of diagnosis and treatment) and apply a monthly probability of OPV infection depending on poliovirus vaccine use, age, diagnosis status, IVIG treatment status and serotype. We randomly and independently sample the serotypes (with a small possibility of two concurrent serotypes) of primary OPV infection, whereas for secondary OPV infection we randomly sample only one serotype. Finally, we determine the monthly progression of OPV infection depending on the long-term poliovirus excreter status determined at birth, and we apply a monthly probability of developing iVAPP while OPV infected, with an income-level-dependent probability that the iVAPP will lead to death. To characterise the updated global iVDPV prevalence behaviour we run 1000 stochastic iterations of the DES model and then aggregate the results.

## Results

Table 3 lists our updated database of global iVDPV excreters and isolations of suspected iVDPVs (or aVDPVs given their unknown source) suggesting prolonged excretion detected to date and cross-referenced between multiple sources and studies [19, 20, 45, 46]. Table 3 includes 143 individuals (120 prolonged, 7 chronic, 15 <6 months and 1 unknown) identified between 1962 and 2018, of which 22 (15%) presented with CVID, while 123 (85%) presented with oPIDs. In the current data, 7 of the 22 individuals (32%) with CVID and none of the 123 individuals with oPIDs met the criteria for chronic excretion. In the column showing the estimated VP1 divergence, we note the individuals for which the estimated duration of iVDPV excretion calculated based on the maximum VP1 divergence exceeds the age of the individual. These cases highlight some of the challenges arising from the use of imperfect information about dose history, inference about the duration of infection from genetic data and the reality that individuals can become infected by viruses in the community. Although we attempted to verify all of the US cases, we could not locate some of the epidemiological and/or virology historical records, and consequently, we most likely missed a small number of iVDPV excreters prior to 2000 (i.e. before recognition of VDPVs). Notably, the definitions of what qualified as a VDPV changed over time for VDPV2, and this impacted the recognition of iVDPV cases.

**Table 3.** Documented iVDPV excreters and isolations of aVDPVs suggesting prolonged excretion for 1962–2018 (as reported by mid-2019)

Year of paralysis onset or first sample collection	Country	Gender	Immune deficiency	Paralysis (yes/no)	Serotype	Age (years) at paralysis onset or first sample collection	Maximum VP1 divergence (%)	Estimated duration of iVDPV excretion (years) based on maximum VP1 divergence	Excreter type	Outcome	Sources
Documented iVDPV excreters											
1962	UK	Male	HGG	No	1	3.0	2.5	2.3	Prolonged	Dead	[19, 20]
1962	UK	Female	HGG	No	3	20.0	2.3	2.1	Prolonged	Dead	[19, 20]
1977	Japan	Male	AGG	Yes	2	1.9	Unknown	Unknown	Prolonged	Dead	[19, 20]
1980	USA	Female	AGG	Yes	2	1.7	1.3	1.2	Prolonged	Dead	[19, 20]
1981	USA	Male	CVID	Yes	1	17.0	10.0	9.1	Chronic	Dead	[19, 20]
1986	USA	Male	XLA	Yes	2	0.9	2.0	1.8	Prolonged	Alive and stopped excreting	[19, 20]
1986; 1992	USA	Female	CVID	No	1; 2	11.0	5.4; 11.8	10.7	Chronic	Unknown	[19, 20]
1987	UK	Male	CVID	No	2	34.0	4.1	3.7	Prolonged	Alive and stopped excreting	[19, 20]
1989	USA	Female	HGG	Yes	2	0.9	0.9	0.8	Unknown	Dead	[20]
1989	USA	Female	AGG	Yes	1	0.6	1.1	1.0	Prolonged	Unknown	[19, 20]
1990	USA	Female	SCID	Yes	2	1.3	1.8	1.6^a^	Prolonged	Dead	[19, 20]
1990	Germany	Male	CVID	Yes	1	7.0	8.3	7.5	Chronic	Alive and stopped excreting	[19, 20]
1991	USA	Female	CVID	Yes	2	0.7	1.4	1.3	Prolonged	Dead	[19, 20]
1995	USA	Female	SCID	Yes	2	0.3	2.2	2.0	Prolonged	Dead	[19, 20]
1995	UK	Male	CVID	No	2	25.0	17.7	16.1	Chronic	Alive	[19, 20]
1995	Iran	Female	HGG	Yes	2	1.4	2.2	2.0^a^	Prolonged	Dead	[19, 20]
1998	Argentina	Male	XLA	Yes	1	3.0	2.8	2.5	Prolonged	Alive and stopped excreting	[19, 20]
2000	Germany	Female	CVID	Yes	1	24.0	12.1	11.0	Chronic	Dead	[19, 20]
2000	UK	Male	CVID	No	2	13.0	7.4	6.7	Chronic	Alive and stopped excreting	[19, 20]
2000	Italy	Female	AGG	Yes	2	1.7	0.9	0.8	Prolonged	Alive and stopped excreting	[19, 20]
2001	China	Unknown	Unknown	Yes	3	Unknown	1.0	0.9	Prolonged	Unknown	[19]
2001	Taiwan	Male	CVID	Yes	1	8.0	3.5	3.2	Prolonged	Alive and stopped excreting	[19, 20]
2002	Kazakhstan	Female	HGG	Yes	2	2.0	2.3	2.1	Prolonged	Dead	[19, 20]
2002	Kuwait	Female	SCID	No	2	2.0	2.0	1.8	Prolonged	Dead	[19, 20]
2002	UK	Female	ICF syndrome	No	2	1.5	2.5	2.3	Prolonged	Alive and stopped excreting	[19, 20]
2003	Peru	Male	AGG	Yes	2	0.8	1.2	1.1	Prolonged	Alive and stopped excreting	[19, 20]
2003	Thailand	Male	HGG	Yes	2	1.5	2.2	2.0	Prolonged	Alive and stopped excreting	[19, 20]
2005	China	Male	XLA	Yes	2; 3	2.0	4.2; 3.9	3.8	Prolonged	Dead	[19, 20]
2005	Morocco	Male	SCID	Yes	2	1.2	2.2	2.0	Prolonged	Dead	[19, 20]
2005	Saudi Arabia	Unknown	SCID	No	2	0.8	4.4	4.0	Prolonged	Dead	[19, 20]
2005	Syria	Female	HGG	Yes	2	0.5	1.3	1.2^a^	Prolonged	Unknown	[19, 20]
2005	Iran	Male	SCID	Yes	2	0.6	1.5	1.4	Prolonged	Dead	[19, 20]
2005	USA	Female	SCID	No	1	0.6	2.3	2.1^a^	Prolonged	Alive and stopped excreting	[19, 20]
2006	Syria	Male	HCI	Yes	2	0.7	2.2	2.0^a^	Prolonged	Dead	[19, 20]
2006	Tunisia	Male	SCID	No	2	0.9	2.0	1.8^a^	Prolonged	Dead	[19, 20]
2006	Iran	Male	SCID	Yes	2	0.8	2.0	1.8	Prolonged	Dead	[19, 20]
2006	Iran	Male	XLA	Yes	3	1.3	2.1	1.9	Prolonged	Dead	[19, 20]
2007	Kuwait	Female	SCID	Yes	3	0.7	1.2	1.1^a^	Prolonged	Alive and stopped excreting	[19, 20]
2007	Belarus	Male	HGG	Yes	2	3.0	1.9	1.7	Prolonged	Unknown	[19, 20]
2007	Egypt	Female	SCID	Yes	3	0.3	1.1	1.0	<6 months	Dead	[20]
2007	Iran	Male	XLA	Yes	2	0.6	1.2	1.1	Prolonged	Alive and stopped excreting	[19, 20]
2007	Iran	Female	SCID	Yes	1; 2	0.4	2; 1.7	1.8	Prolonged	Dead	[19, 20]
2009	Tunisia	Male	SCID	No	1	7.3	1.2	1.1	Prolonged	Alive and stopped excreting	[19, 20]
2009	USA	Female	CVID	Yes	2	44.0	12.3	11.2	Chronic	Dead	[19, 20]
2009	Argentina	Male	XLA	Yes	1	1.3	4.1	3.7^a^	Prolonged	Alive and stopped excreting	[19, 20]
2009	Colombia	Male	AGG	Yes	2	1.3	1.5	1.4	Prolonged	Unknown	[19, 20]
2009	India	Male	CVID	Yes	1	11.0	5.2	4.7	Prolonged	Dead	[19, 20]
2010	Sri Lanka	Male	SCID	No	2	0.8	1.3	1.2^a^	Prolonged	Dead	[19, 20]
2010	India	Female	CVID	Yes	2	10.0	1.6	1.5	Prolonged	Alive and stopped excreting	[19, 20]
2010	Algeria	Female	HLA-DR	Yes	2	0.5	1.8	1.6^a^	Prolonged	Dead	[19, 20]
2010	Iraq	Male	PID	Yes	2	0.7	1.2	1.1^a^	Prolonged	Dead	[19, 20]
2011	Algeria	Female	HLA-DR	Yes	3	1.2	2.8	2.5	Prolonged	Alive and stopped excreting	[19]
2011	Algeria	Female	HLA-DR	No	3	0.3	1.2	1.1	<6 months	Alive and stopped excreting	WHO^b^
2011	Algeria	Male	HLA-DR	No	2	0.8	2.6	2.4^a^	Prolonged	Dead	[19]
2011	China	Male	CVID	No	2	0.6	0.9	0.8	<6 months	Alive	WHO^b^
2011	Egypt	Female	SCID	No	2	0.7	1.4	1.3^a^	Prolonged	Dead	[19, 20]
2011	India	Male	HGG	Yes	2	1.0	0.8	0.7	Prolonged	Dead	[19]
2011	India	Female	HGG	Yes	2	0.3	0.6	0.5^a^	<6 months	Dead	WHO^b^
2011	India	Male	CVID	Yes	3	7.0	1.2	1.1	Prolonged	Alive and stopped excreting	[19]
2011	Sri Lanka	Female	CVID	Yes	3	8.4	2.6	2.4	Prolonged	Alive and stopped excreting	[19, 20]
2011	Turkey	Male	CVID	No	2	1.0	1.8	1.6^a^	Prolonged	Unknown	[19, 20]
2011	West Bank and Gaza Strip	Male	SCID	No	2	1.0	1.2	1.1	Prolonged	Dead	[19, 20]
2011	China	Male	CVID	Yes	3	2.3	3.3	3.0	Prolonged	Dead	[19, 20]
2011	China	Female	CVID	Yes	2	9.0	1.9	1.7	Prolonged	Dead	[19, 20]
2011	Egypt	Male	SCID	Yes	3	0.5	5.3	4.8	Prolonged	Alive and stopped excreting	[19, 20]
2011	Egypt	Male	XLA	Yes	1	1.7	2.1	1.9	Prolonged	Alive and stopped excreting	[19, 20]
2011	Iran	Male	CID	Yes	2	0.7	2.1	1.9	Prolonged	Dead	[19, 20]
2011	Iran	Male	XLA	Yes	2	1.3	3.8	3.4	Prolonged	Alive and stopped excreting	[19, 20]
2011	South Africa	Male	XLA	Yes	3	0.8	1.9	1.7^a^	Prolonged	Alive and stopped excreting	[19, 20]
2011	Iran	Male	SCID	Yes	1; 2	2.1	2.7; 3.3	3.0	Prolonged	Dead	[19, 20]
2012	Egypt	Male	SCID	Yes	1; 2	0.3	2.66; 3.3	3.0^a^	<6 months	Dead	WHO^b^
2012	India	Female	HGG	Yes	2	0.5	2.0	1.8^a^	Prolonged	Dead	[19, 20]
2012	China	Male	CVID	Yes	2; 3	0.9	1.7; 2.3	1.5^a^	Prolonged	Alive and stopped excreting	[19, 20]
2012	Iran	Male	SCID	Yes	2	0.5	2.4	2.2	Prolonged	Dead	[19, 20]
2012	Egypt	Female	SCID	No	2	0.5	1.1	1.0^a^	<6 months	Dead	[20]
2012	Iraq	Male	PID	Yes	2	2.0	2.4	2.2	Prolonged	Dead	[19, 20]
2012	Iran	Male	XLA	Yes	2	1.0	1.5	1.4	Prolonged	Alive and stopped excreting	[19, 20]
2012	Egypt	Male	SCID	Yes	2	0.4	1.0	0.9	Prolonged	Dead	[19, 20]
2013	India	Male	CVID	Yes	2	0.9	0.7	0.6	Prolonged	Alive and stopped excreting	[19]
2013	Algeria	Female	HLA-DR	No	2	0.4	1.5	1.4^a^	Prolonged	Alive	[19]
2013	China	Male	PID	Yes	2; 3	0.6	0.6; 2.1	1.9^a^	Prolonged	Dead	[19, 20]
2013	Egypt	Female	SCID	No	2	0.5	1.3	1.2	Prolonged	Dead	[19]
2013	Libya	Female	SCID	No	2	0.4	1.0	0.9	Prolonged	Alive and stopped excreting	[19, 20]
2013	India	Male	CVID	Yes	2	0.6	0.9	0.8	Prolonged	Dead	[19, 20]
2013	India	Male	HGG	Yes	2	0.8	0.7	0.6	Prolonged	Dead	[19, 20]
2013	Saudi Arabia	Female	SCID	No	2	2.5	4.4	4.0	Prolonged	Alive and stopped excreting	[19, 20]
2013	Iran	Male	PID	Yes	2	1.1	0.9	0.8	Prolonged	Dead	[19, 20]
2013	USA	Male	SCID	Yes	1	0.6	1.3	1.2^a^	Prolonged	Dead	[19, 20]
2013	Afghanistan	Male	Febrile neutropenia ID	Yes	2	3.0	0.9	0.8	Prolonged	Alive and stopped excreting	[19, 20]
2014	Iran	Male	XLA	Yes	1	0.8	1.8	1.6	Prolonged	Alive and stopped excreting	[19, 20]
2014	Tunisia	Male	SCID	No	2	11.8	1.0	0.9	<6 months	Alive and stopped excreting	[20]
2014	Iran	Male	AGG	Yes	2	0.7	1.9	1.7	Prolonged	Alive and stopped excreting	[19, 20]
2014	Iran	Male	SCID	No	1	0.8	3.3	3.0	Prolonged	Alive and stopped excreting	[19, 20]
2014	Turkey	Female	SCID	No	3	2.0	1.7	1.5	Prolonged	Alive and stopped excreting	[19, 20]
2014	China	Male	PID	Yes	3	1.2	1.4	1.3^a^	Prolonged	Alive and stopped excreting	[19, 20]
2014	Albania	Male	XLA	Yes	3	0.4	1.0	0.9	Prolonged	Alive and stopped excreting	[19, 20]
2015	China	Male	Unknown	Yes	2	0.7	0.8	0.7	Prolonged	Alive	[19, 20]
2015	China	Male	Unknown	Yes	2	2.2	2.4	2.2	Prolonged	Alive	[20]
2015	West Bank and Gaza Strip	Female	SCID	No	2	0.5	1.4	1.3	Prolonged	Alive and stopped excreting	[19, 20]
2015	India	Female	CVID	Yes	2	2.0	3.6	3.3	Prolonged	Alive and stopped excreting	[19, 20]
2015	Iran	Female	SCID	Yes	2	0.5	1.6	1.5^a^	Prolonged	Dead	[19, 20]
2015	Iraq	Female	Unknown	Yes	2	0.8	1.7	1.5^a^	Prolonged	Dead	[19, 20]
2015	Iran	Male	SCID	No	2	0.3	0.7	0.6^a^	<6 months	Dead	[20]
2015	Iran	Female	SCID	No	2	1.0	1.1	1.0	Prolonged	Dead	[19, 20]
2015	Iran	Male	SCID	No	2; 3	1.0	2.4; 2.7	2.5^a^	Prolonged	Alive	[19, 20]
2015	Nigeria	Female	Unknown	Yes	2	0.1	0.7	0.6	<6 months	Dead	[20]
2015	Iran	Female	SCID	No	2	0.8	3.2	2.9	Prolonged	Alive	[19, 20]
2015	Oman	Male	SCID	No	2	0.7	1.6	1.5^a^	Prolonged	Dead	[19, 20]
2015	Algeria	Male	Antibody and T-cell disorder	No	2	0.8	1.7	1.5^a^	Prolonged	Dead	[19, 20]
2015	Turkey	Unknown	Unknown	No	3	Unknown	1.7	1.5	Prolonged	Unknown	[20]
2015	Turkey	Unknown	Unknown	Yes	2	Unknown	0.8	0.7	Prolonged	Unknown	[20]
2015	India	Male	SCID	No	3	4.0	10.2	9.3^a^	Prolonged	Alive and stopped excreting	[20, 51]
2015	Egypt	Male	XLA	Yes	2	1.0	1.9	1.7^a^	Prolonged	Alive and stopped excreting	[19, 20]
2016	Egypt	Male	SCID	No	2	1.0	1.4	1.3	Prolonged	Alive and stopped excreting	[19, 20]
2016	India	Male	XLA	Yes	2	5.3	0.6	0.5	Prolonged	Alive and stopped excreting	[19]
2016	Iraq	Female	Unknown	Yes	2	0.6	0.7	0.6	Prolonged	Dead	[20, 45]
2016	Argentina	Male	AGG	No	2	0.9	0.9	0.8	<6 months	Alive and stopped excreting	[45]
2016	Egypt	Female	SCID	No	2	Unknown	0.7	0.6	<6 months	Alive and stopped excreting	[20, 45]
2016	Pakistan	Male	Unknown	Yes	2	0.6	1.1	1.0^a^	Prolonged	Unknown	[45]
2016	Tunisia	Female	HLA-DR	Yes	3	0.6	1.0	0.9^a^	Prolonged	Dead	[45]
2016	West Bank and Gaza Strip	Male	SCID	No	2	0.7	1.0	0.9	Prolonged	Dead	[45, 52]
2016	Nigeria	Male	Unknown	Yes	2	2.0	0.9	0.8	Prolonged	Unknown	[45]
2016	Iran	Male	AGG	Yes	2	1.2	0.7	0.6	Prolonged	Alive and stopped excreting	[45]
2017	Egypt	Male	SCID	Yes	2	1.0	1.9	1.7	<6 months	Dead	[45]
2017	Iran	Male	PID	No	3	1.2	1.3	1.2	Prolonged	Unknown	[45]
2017	Turkey	Female	CID	No	3	0.4	1.1	1.0^a^	<6 months	Unknown	WHO^b^
2017	Egypt	Male	XLA	Yes	3	1.3	2.0	1.8	Prolonged	Alive	[46]
2017	Egypt	Female	SCID	Yes	1	2.0	2.4	2.2	Prolonged	Dead	[46]
2017	China	Male	Unknown	Yes	3	1.0	0.8	0.7	Prolonged	Alive	[46]
2017	West Bank and Gaza Strip	Female	SCID	No	3	0.5	1.8	1.6	<6 months	Alive and stopped excreting	[46]
2018	Colombia	Female	Unknown	Yes	1	0.9	1.4	1.3	Prolonged	Unknown	[46]
2018	Iran	Female	Unknown	Yes	1	1.1	3.4	3.1	Prolonged	Alive	WHO^b^
2018	Egypt	Unknown	CID	No	1	0.8	1.7	1.5	Prolonged	Alive	WHO^b^
2018	China	Male	Unknown	Yes	3	1.0	1.1	1.0	Prolonged	Unknown	[46]
2018	China	Male	Unknown	Yes	3	0.8	1.4	1.3	Prolonged	Unknown	[46]
2018	South Africa	Male	AGG	Yes	3	0.2	1.6	1.5	Prolonged	Alive	[46]
2018	Egypt	Female	CID	Unknown	3	1.3	1.6	1.5	Prolonged	Alive and stopped excreting	WHO^b^
2018	Egypt	Male	CID	No	1	0.8	1.7	1.5	Prolonged	Alive	WHO^b^
2018	Egypt	Female	SCID	Yes	1	1	2.6	2.4	<6 months	Dead	WHO^b^
2018	Egypt	Female	SCID	No	1	0.8	0.6	0.5	Prolonged	Alive	WHO^b^
2018	Egypt	Male	SCID	No	3	1.2	1.6	1.5	Prolonged	Alive	WHO^b^
2018	Iran	Female	PID	No	1	0.6	1	0.9	Prolonged	Dead	WHO^b^
2018	Iran	Male	B-cell deficiency	Yes	1	1	1.6	1.5	Prolonged	Alive	WHO^b^
Isolations of aVDPVs suggesting prolonged excretion											
1998–2008	Israel	Unknown	Unknown	Unknown	2	Unknown	15.1	13.7	Unknown	Unknown	
2006–2007	Israel	Unknown	Unknown	Unknown	2	Unknown	8.8	8.0	Unknown	Unknown	
2002	Estonia	Unknown	Unknown	Unknown	3	Unknown	13.3	12.1	Unknown	Unknown	
2003	Slovakia	Unknown	Unknown	Unknown	2	Unknown	15.0	13.6	Unknown	Unknown	
2008–2010	Finland	Unknown	Unknown	Unknown	1, 2, 3	Unknown			Unknown	Unknown	
2017	Australia	Unknown	Unknown	Unknown	2	Unknown			Unknown	Unknown	

AGG, agammaglobulinemia; iVDPV, immunodeficiency-related vaccine-derived poliovirus; CID, combined immune deficiency; CVID, common variable immunodeficiency disease; ID, immunodeficiency disease; HGG, hypogammaglobulinemia; HLA, human leukocyte antigen; ICF, immunodeficiency, centromeric region instability, facial anomalies; PID, primary immunodeficiency diseases; SCID, severe combined immunodeficiency disease; XLA, X-linked agammaglobulinemia; VP1, viral protein 1; WHO, World health Organisation.

a Estimated duration of iVDPV excretion based on maximum VP1 divergence exceeds the age of patient.

b WHO, personal communication.

Table 4 presents model estimates of global diagnosed CVID and oPID prevalence in January 2011, 2013, 2015 and 2018, which increased from 8326 and 13 660 to 10 494 and 18 868, respectively. These results fall within ~1600 patients compared to the number of patients suggested by the JMFN surveys (which increased from 7613 and 12 073 to 11 996 and 18 805, respectively, as shown in Table 1). For 2011–2018, the model estimates the incidence of 63 iVAPP cases and six chronic excreters compared to 56 known iVAPP cases, one chronic excreter and multiple environmental aVDPV isolates that suggest at least three possible chronic excreters in this period of time (Table 3). We note that delays in reporting of iVDPV cases may lead to higher incidence for the 2011–2018 period at a later point in time, particularly for the more recent years (e.g. our previous study [7] reported 26 iVAPP cases for 2009–2013 known at the time of its publication, but this update reports 34 iVAPP cases for that same period). Overall, we observe an increase in the estimated prevalence over time (compared to our previous study [7]), which we largely attribute to updates in our assumptions about survival and later timing for when some of the 72 blocks stopped or will stop using OPV.

**Table 4.** Diagnosed CVID and oPID prevalence in the model

Number of	2011	2013	2015	2018
Diagnosed CVID	8326	8903	9523	10 494
Diagnosed oPID	13 660	15 087	16 598	18 868
Treated CVID and oPID (IVIG)	15 019	16 798	18 526	20 781

CVID, common variable immune deficiency; IVIG, intravenous immunoglobulin; oPID, other PID with B-cell involvement relevant to long-term poliovirus excretion; PID, primary immune deficiency.

**Fig. 3.**
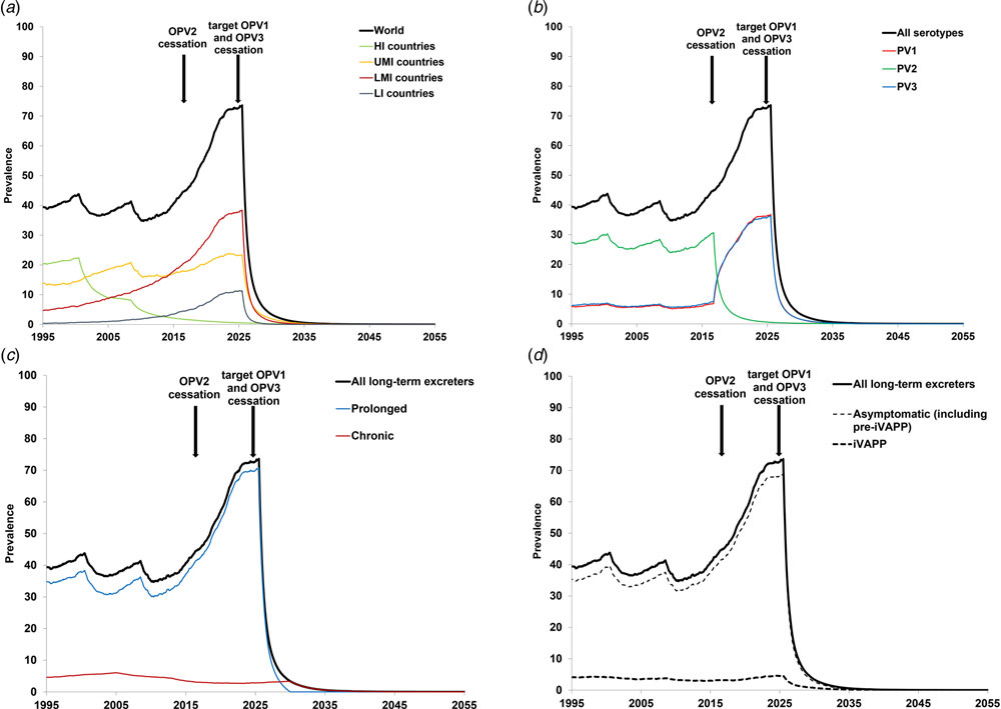
Prevalence of long-term iVDPV excreters in the absence of PAVD use, based on the monthly averages of 1000 iterations of the DES model.

Figure 3 shows the baseline prevalence (i.e. without PAVDs) of long-term iVDPV excreters. Figure 3a shows the prevalence by the income level, which suggests that middle-income countries account for most of the current long-term excreters, while the prevalence in high-income countries continues to decline steadily since these countries stopped all OPV use. Figure 3b shows the impact of stopping serotype 2 containing OPV use (i.e. OPV2 cessation) and assumptions about serotype-specific infections (i.e. a shift to first-infections of individuals with serotypes 1 and 3). Notably, Figure 3b suggests a sharp drop in long-term serotype 2 excreters, but a simultaneous rise in serotype 1 and serotype 3 excreters, caused by the new assumed distribution between serotypes 1 and 3 for the probability of the first OPV infection. Chronic excreters amount to 4–16% of all long-term excreters, most of them residing in HI countries. After the expected global cessation of all OPV use, the prevalence of prolonged excreters drops quickly, while a few chronic excreters continue to exist for over a decade (see Fig. 3c). Only up to 11% of long-term excreters present with iVAPP, while the remainder either recover or die before the paralysis occurs (see Fig. 3d).

## Discussion

With the GPEI already beginning its transition of activities to countries and other decentralised components of its partner organisations, developing plans for long-term risk management requires attention. Despite the continued circulation of WPV1 in Pakistan and Afghanistan[53], the GPEI aspiration to stop all OPV use and successfully maintain a polio-free world continues to require a longer time horizon and additional resources. This updated analysis of iVDPV risk, despite considerable uncertainty, confirms prior findings [4, 7] that iVDPVs may pose risks that require management after global OPV cessation. Although PID patient screening generally improves with time [41–44], the actual prevalence and the proportion of PID patients who may develop prolonged or chronic excretion (i.e. CVID and oPIDs) and the extent to which health systems will identify and treat these patients remains highly uncertain [7, 17]. Our estimates of current long-term excreter prevalence remain higher than reported, as expected given the lack of a comprehensive surveillance system, although the gap should continue to decrease as health systems increasingly expand their capacity to find individuals with PIDs.

Our model remains limited to the assumptions we make based on the insufficient data and high uncertainty around characteristics of PID patients and their relation to iVDPV excretion. Uncertainty about the transmissibility and neurovirulence of viruses re-introduced into populations from iVDPV excreters leads to significant uncertainty about the potential value of developing PAVDs [17]. We combine all non-CVID defects into one homogeneous oPID category despite the differences that exist between them, but as research reveals more evidence about PIDs, this may prove overly simplistic. Insufficient information about their survival and probability of long-term infection (given OPV exposure) led us to a conservative approach of assuming equal probability of becoming prolonged excreter for all oPID patients, which may overestimate prolonged excretion in the model.

The results of our model strongly depend on the assumption and timing of successful cessation of the remaining serotypes. Cessation of all OPV will eventually stop the creation of new long-term and chronic excreters, however it will take time for the existing chronic excreters to clear their infections or die. In the context of discussions related to potentially stopping serotype 3 OPV use (i.e. OPV3 cessation) before stopping serotype 1 OPV (OPV1), the results in Figure 3 would change with the die-out of serotype 3, the shift of all iVDPV risk to serotype 1, and the timing of OPV1 cessation. In Figures 3a, c and d, changing the time of the last OPV cessation will change the timing of the dramatic drop to shortly after the end of the last OPV use (i.e. OPV1 cessation), with the totals expected to continue to increase with time until cessation due to increases in population, PID diagnosis, treatment and survival. In Figure 3b, the blue curve would drop shortly after OPV3 cessation and the red curve would jump up to the level of the black curve, because then all first-infections with OPV would occur with OPV1 (based on our explicit assumption of no inherently lower likelihood of iVDPV from OPV1). In our model, the total (‘all serotypes’) would continue to increase until the last OPV cessation. In addition to these possibilities, OPV2 cessation did not go as smoothly as hoped, and the use of monovalent OPV2 continues to date (as of August 2019) in response to cVDPV2 outbreaks[54]. This means that the model should include the possibility of creating some new iVDPV2 excreters in the areas of serotype 2 outbreaks. In the event of needing to restart OPV2 use globally[54], [55], the risks of iVDPV excreters will change due to the exposure of CVID and oPID individuals again potentially becoming first-infected with OPV2. We emphasise that the risks from iVDPV excreters only become recognisable once OPV cessation occurs, and while countries use OPV, the risks from VAPP and cVDPV outbreaks dominate.

In the absence of tools for treating iVPDV excreters, performing surveillance to identify them becomes challenging. However, once PAVDs become available, developing strategies to cost-effectively identify and treat any long-term iVDPV excreters remaining after OPV cessation will help to prevent iVAPP in these individuals and to reduce or eliminate them as a potential source for re-introduction of poliovirus in a world with significantly lowered and decreasing levels of population immunity to poliovirus transmission.
